# Supplementary material for: Complex‐centric proteome profiling by SEC‐SWATH‐MS
Source: Mol Syst Biol. 2019 Jan 14;15(1):e8438. doi: 10.15252/msb.20188438 (PMC6346213; doi:10.15252/msb.20188438)
Supplement: Supplementary file 8 — Dataset EV7 [file MSB-15-e8438-s008.zip › feature_plots_string/O75691.pdf]

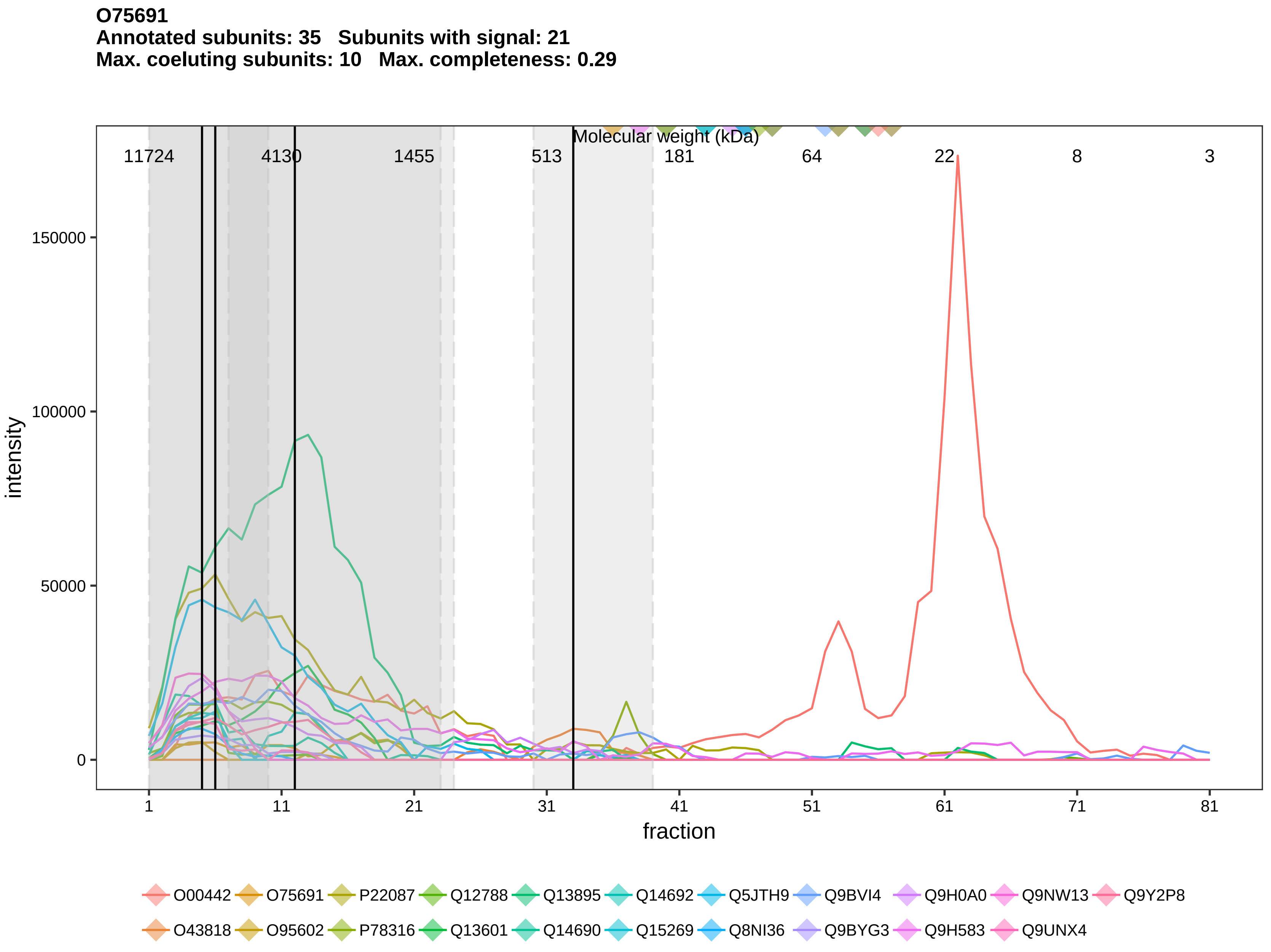
O75691
Annotated subunits: 35 Subunits with signal: 21
Max. coeluting subunits: 10 Max. completeness: 0.29
Molecular weight (kDa)
11724
4130
1455
513
181
64
22
8
3
150000
100000
50000
0
intensity
1
11
21
31
41
51
61
71
81
fraction
O00442
O75691
P22087
Q12788
Q13895
Q14692
Q5JTH9
Q9BVI4
Q9H0A0
Q9NW13
Q9Y2P8
O43818
O95602
P78316
Q13601
Q14690
Q15269
Q8NI36
Q9BYG3
Q9H583
Q9UNX4
